# Assessing the Diagnostic Accuracy of BiomedCLIP for Detecting Contrast Use and Esophageal Strictures in Pediatric Radiography †

**DOI:** 10.3390/jcm15031150

**Published:** 2026-02-02

**Authors:** Artur Fabijan, Michał Kolejwa, Agnieszka Zawadzka-Fabijan, Robert Fabijan, Róża Kosińska, Emilia Nowosławska, Anna Socha-Banasiak, Natalia Lwow, Marcin Tkaczyk, Krzysztof Zakrzewski, Elżbieta Czkwianianc, Bartosz Polis

**Affiliations:** 1 Department of Neurosurgery, Polish-Mother’s Memorial Hospital Research Institute, 93-338 Lodz, Poland; roza.w.kosinska@gmail.com (R.K.); emilia.nowoslawska@iczmp.edu.pl (E.N.); krzysztof.zakrzewski@iczmp.edu.pl (K.Z.); jezza@post.pl (B.P.); 2 Department of Gastroenterology, Allergology and Pediatrics, Polish Mother’s Memorial Hospital Research Institute, 93-338 Lodz, Poland; michal.kolejwa@umed.lodz.pl (M.K.); sochabanasiak@gmail.com (A.S.-B.); natalia.lwow@umed.lodz.pl (N.L.); elcia@friend.pl (E.C.); 3 Department of Pediatrics, Immunology and Nephrology, Medical University of Lodz, 90-419 Lodz, Poland; marcin.tkaczyk@umed.lodz.pl; 4 Department of Medical Rehabilitation, Faculty of Health Sciences, Medical University of Lodz, 90-419 Lodz, Poland; agnieszka.zawadzka@umed.lodz.pl; 5 Independent Researcher, Luton LU2 0GS, UK; robert.f.fabijan@gmail.com

**Keywords:** vision–language models, BiomedCLIP, pediatric radiology, esophageal stricture detection, artificial intelligence

## Abstract

**Background/Objectives**: Vision–language models such as BiomedCLIP are increasingly investigated for their diagnostic potential in medical imaging. Although these foundation models show promise in general radiographic interpretation, their application in pediatric domains—particularly for subtle, postoperative findings like esophageal strictures—remains underexplored. This study aimed to evaluate the diagnostic performance of BiomedCLIP in classifying pediatric esophageal radiographs into three clinically relevant categories: presence of contrast agent, full esophageal visibility, and presence of esophageal stricture. **Methods**: We retrospectively analyzed 143 pediatric esophageal X-rays collected between 2021 and 2025. Each image was annotated by two pediatric radiology experts and categorized according to esophageal visibility, contrast presence, and stricture occurrence. BiomedCLIP was used in a zero-shot classification setup without fine-tuning. Model predictions were converted into binary outcomes and assessed against the ground truth using a comprehensive suite of 27 performance metrics, including accuracy, sensitivity, specificity, F1-score, AUC, and calibration analyses. **Results**: BiomedCLIP achieved high precision (88.7%) and a favorable AUC (85.4%) in detecting contrast agent presence, though specificity remained low (20%), leading to a high false-positive rate. The model correctly identified all cases of non-visible esophagus, but was untestable in predicting full visibility due to the absence of positive cases. Critically, its performance in detecting esophageal strictures was poor, with accuracy at 24%, sensitivity at 44%, specificity at 18%, and AUC of 0.26. Statistical overlap between contrast and stricture predictions indicated a lack of semantic differentiation within the model’s latent space. **Conclusions**: BiomedCLIP shows potential in detecting high-salience features such as contrast but fails to reliably identify esophageal strictures. Limitations include class imbalance, absence of fine-tuning, and architectural constraints in recognizing subtle morphologic abnormalities. These findings emphasize the need for domain-specific adaptation of foundation models before clinical implementation in pediatric radiology.

## 1. Introduction

Surgical management of pediatric esophageal conditions relies heavily on perioperative and postoperative decision-making rather than intraoperative findings alone. In routine clinical practice, assessment of surgical outcomes and detection of postoperative complications—such as anastomotic strictures—are predominantly based on serial radiographic examinations performed during follow-up, making imaging interpretation a central component of postoperative care. In recent years, artificial intelligence has been increasingly explored as a supportive tool in surgical settings, with growing evidence that it may enhance screening efficiency, diagnostic accuracy, and clinical decision support across a range of surgical diseases [1].

Within radiology, foundation models capable of multimodal and multitask reasoning have emerged as particularly promising, owing to the structured nature of imaging data and the field’s early adoption of AI technologies. However, despite strong technical performance reported for several general-purpose and biomedical foundation models, their clinical readiness remains uncertain, with documented limitations related to safety, external validation, and performance in edge-case or highly specialized scenarios [2]. Importantly, recent comparative studies suggest that while foundation-model embeddings can be highly effective when paired with task-specific adapters or supervised optimization, their standalone performance without adaptation—especially for fine-grained radiographic tasks—may be substantially lower, including for biomedical models such as BiomedCLIP [3]. Against this background, the present study aims to critically evaluate the real-world diagnostic performance and limitations of a biomedical vision–language foundation model applied in a strictly zero-shot setting to pediatric esophageal radiography.

Open-source artificial intelligence models (OSAIM) are freely accessible tools increasingly used in computer science and medicine, where they support advances in diagnostics and treatment planning [4,5,6,7,8]. Although powerful in visual data interpretation, most are not tailored for medical imaging, such as X-rays. Models like BiomedCLIP address this limitation by combining natural language processing with medical image analysis, expanding their utility in diagnostic support and therapeutic decision-making [9]. Growing interest in clinical applications highlights their potential in radiology and broader medical practice.

Over the past year, global interest in open-source artificial intelligence models (OSAIMs) has significantly increased, driven in part by the emergence of advanced language models such as ChatGPT, developed by OpenAI. Among OpenAI’s notable contributions is the Contrastive Language–Image Pretraining (CLIP) model, which integrates natural language understanding with visual data processing. CLIP supports a wide range of vision–language tasks, including zero-shot image classification, image captioning, and visual question answering [10]. This versatility stems from its training on a diverse dataset comprising image–text pairs, enabling it to learn a shared multimodal embedding space that facilitates efficient interpretation of both modalities.

Building upon CLIP, several domain-specific adaptations have emerged. Notably, SDA-CLIP has demonstrated efficacy in surgical task recognition across varying environments, such as simulations and real-world procedures [11]. Similarly, SleepCLIP has adapted CLIP’s architecture to the domain of sleep staging, suggesting potential improvements in the diagnosis of sleep-related disorders [12].

**Figure 1:**
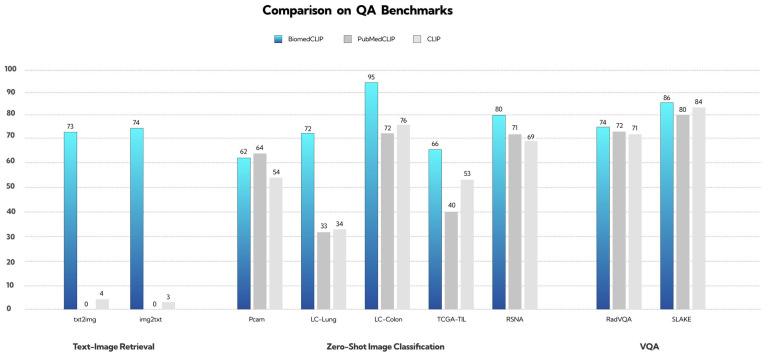
A comparative analysis of BiomedCLIP, PubMedCLIP, and the standard CLIP model across text–image retrieval, zero-shot image classification, and visual question answering (VQA) benchmarks demonstrates clear performance distinctions. The reproduced results show that BiomedCLIP consistently outperforms the other models, particularly in clinical and biomedical tasks, where it achieves substantially higher accuracy. These findings highlight the model’s strengthened ability to integrate visual and textual information and its strong suitability for medical imaging applications. BiomedCLIP-PubMedBERT_256-vit_base_patch16_224 Available online: https://huggingface.co/microsoft/BiomedCLIP-PubMedBERT_256-vit_base_patch16_224 (accessed on 2 September 2025).

One of the most prominent biomedical adaptations is BiomedCLIP (formally Biomed-CLIP-PubMedBERT_256-vit_base_patch16_224), a vision–language foundation model that merges natural language processing with medical image analysis. Trained on the extensive PMC-15M dataset, which contains 15 million image–description pairs sourced from PubMed Central, BiomedCLIP is capable of interpreting both clinical texts and diagnostic images (Figure 1) [13].

The model’s architecture integrates PubMedBERT, a language model optimized for biomedical text, with a Vision Transformer tailored for medical imaging. This configuration allows BiomedCLIP to achieve state-of-the-art performance across various benchmark datasets. Its applications are diverse and include cross-modal retrieval, medical image classification, and visual question answering. Owing to its robust multimodal capabilities, BiomedCLIP is increasingly recognized as a valuable tool in diagnostic support, clinical research, and medical education [13].

Despite its strengths, BiomedCLIP exhibits limitations on certain benchmarks. For example, in the PCam dataset, PubMedCLIP outperformed BiomedCLIP, achieving higher classification accuracy. This highlights the importance of continued model optimization and suggests that domain-specific datasets may benefit from more narrowly tailored approaches.

In our recent study, we compared BiomedCLIP and ChatGPT-4o in identifying spinal stabilization systems on pediatric posturographic X-ray images. While GPT-4o demonstrated high overall accuracy and superior performance in complex classifications, BiomedCLIP showed perfect consistency in binary detection but struggled with detailed system differentiation [14]. In a previous investigation, we assessed BiomedCLIP’s ability to classify scoliosis in pediatric X-rays, finding strong performance in detecting severe cases. However, its accuracy declined for milder curves and when distinguishing between single- and double-curve scoliosis, highlighting the need for further model refinement [9].

Esophageal atresia (EA) is a rare but serious congenital malformation of the gastrointestinal tract, which is characterized by an interruption of the continuity of the esophagus, often associated with tracheoesophageal fistula. Esophageal atresia occurs with an incidence of approximately 1:2500–1:4500 live births [15]. No clear sex-related differences have been reported, although some studies suggest a slight predominance in males [16]. In about 50% of patients, additional congenital anomalies coexist, most frequently within the VACTERL (V—Vertebral anomalies, A—Anal atresia, C—Cardiac defects, TE—Tracheo-Esophageal fistula, R—Renal anomalies, L—Limb defects) association or trisomy syndromes [17].

Suspicion of esophageal atresia can already be raised prenatally on ultrasound, when polyhydramnios and an absent or very small stomach bubble are observed. The final diagnosis, however, is made after birth, when feeding difficulties, excessive salivation and episodes of coughing or cyanosis appear [16,18]. Early diagnosis is critical to prevent aspiration-related complications.

**Figure 2:**
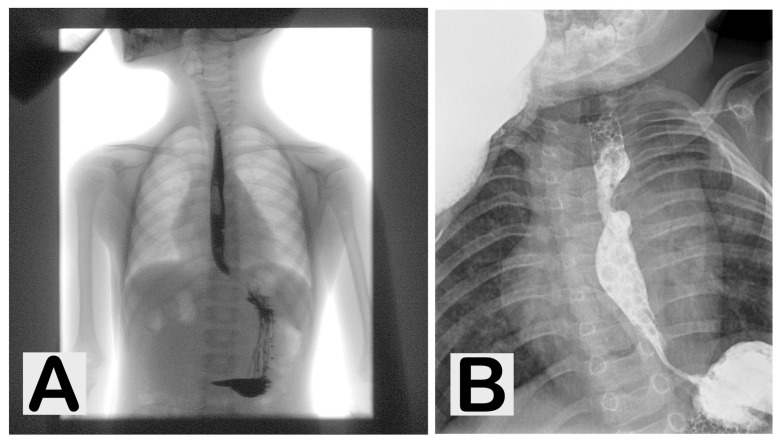
Contrast-enhanced esophagograms in pediatric patients. (**A**) Normal esophageal transit with continuous contrast column and no evidence of stricture or dilation. (**B**) Radiographic image showing esophageal stricture with proximal dilation and impaired passage of contrast medium, suggestive of an anastomotic complication.

An important role in confirming the diagnosis is played by radiography. Contrast-enhanced radiographic studies are especially useful in doubtful cases or when there is a suspicion of tracheoesophageal fistula, as they allow assessment of oesophageal continuity and the site of obstruction. Radiology also has a significant role in postoperative monitoring, particularly in detecting anastomotic complications (Figure 2) [19,20].

Newborns with EA typically present with difficulties in swallowing, choking during feeding, and—if tracheoesophageal fistula is present—recurrent respiratory infections [21]. These symptoms appear immediately after the initiation of feeding.

The standard treatment is surgical reconstruction of the esophagus. Surgery is usually performed within the first days of life [18,22]. In cases with a wide gap between the oesophageal ends, delayed reconstruction or oesophageal replacement with intestinal segments may be required [17].

One of the most frequent complications following surgical repair of EA is the development of anastomotic strictures, which may occur in up to 40% of patients. These strictures can cause recurrent dysphagia, regurgitation and feeding difficulties, often requiring repeated endoscopic balloon dilatations [19,23]. Contrast oesophagography is a valuable tool in diagnosing these strictures, allowing both localisation and assessment of the degree of narrowing, as well as ruling out other structural problems [20].

Despite advances in surgical and neonatal intensive care, patients after EA repair require long-term follow-up. The most common complications include anastomotic strictures requiring endoscopic dilatation [18,19,23], gastroesophageal reflux disease [24], oesophageal dysmotility [16], as well as recurrent respiratory infections and chronic airway obstruction [16,21]. Long-term studies also indicate reduced quality of life in some patients due to persistent dysphagia and reflux [25].

Applying AI in pediatric radiology presents unique challenges due to limited data and the distinct variability in pediatric anatomy and diseases [26]. Consequently, the adoption of AI for children’s imaging has lagged behind adult applications—for instance, as of 2024, only about 8% of FDA-cleared radiology AI tools are applicable to pediatric cases, with fewer than 1% specifically designed for pediatric use [27]. Nonetheless, initial efforts show promise. Xie et al. developed a machine learning model to predict refractory esophageal strictures after esophageal atresia repair, enabling early identification of high-risk patients [28]. This result suggests that AI can assist in postoperative monitoring by detecting which children are likely to develop significant strictures, illustrating the potential benefit of tailored pediatric AI solutions in rare conditions.

Moreover, zero-shot learning (ZSL) has emerged as a valuable strategy for tackling imbalanced datasets and rare diseases. By leveraging semantic knowledge from large vision–language models, ZSL algorithms can recognize previously unseen or under-represented classes without dedicated training data [29]. Recent studies demonstrate that ZSL can accurately distinguish rare disease subtypes, markedly reducing dependence on extensive labeled datasets [30]. This capability is especially relevant for uncommon pediatric disorders such as esophageal atresia, where obtaining large, balanced image datasets is inherently difficult. Incorporating ZSL methods in our approach thus underscores the novelty of this research, as it addresses the ‘long-tail’ problem in pediatric imaging by effectively handling conditions with scarce data.

Esophageal atresia remains a severe congenital anomaly that requires early diagnosis and prompt surgical intervention. Although survival has improved, many patients continue to struggle with chronic complications that affect their quality of life. Long-term monitoring and multidisciplinary care are therefore indispensable. The aim of this study was to evaluate the diagnostic performance of BiomedCLIP in classifying pediatric esophageal X-ray images into three clinically relevant categories: full esophageal visibility, presence of contrast agent, and signs of esophageal stricture. We sought to assess the model’s accuracy and potential clinical utility in detecting key diagnostic features in pediatric radiology.

**Hypotheses** **H1.** *BiomedCLIP will accurately detect the presence of contrast agent in pediatric esophageal X-ray images, demonstrating high sensitivity.*

**Hypotheses** **H2.** *BiomedCLIP will correctly identify esophageal strictures on radiographic images, aligning with expert radiological interpretation.*

## 2. Materials and Methods

### 2.1. Study Design

In this study, we evaluated the performance of BiomedCLIP in classifying pediatric esophageal X-ray images across three predefined binary tasks: (i) esophageal visibility, (ii) presence of contrast agent, and (iii) presence of esophageal stricture. A total of 143 pediatric esophageal radiograms acquired during routine diagnostic procedures were included in the analysis.

Although contrast-enhanced esophagography was the predominant imaging modality in this cohort, not all radiograms demonstrated visible contrast at the time of acquisition or interpretation. Therefore, the presence of contrast agent was treated as a binary classification variable rather than as an inclusion criterion. Of the analyzed images, 128 demonstrated visible contrast within the esophageal lumen, and pediatric radiology experts identified radiographic features consistent with esophageal narrowing in 29 cases.

The dataset consisted of pediatric X-ray images collected between 2021 and 2025 as part of standard clinical care. Only images from patients whose legal guardians provided informed consent for the use of radiographic data for scientific purposes were included.

According to applicable regulations, the study did not require separate ethical approval. The Bioethics Committee, in Opinion No. 58/2021 issued on 21 September 2021, determined that the project did not meet the criteria of a medical experiment and was therefore exempt from ethical review. The study involved a retrospective analysis of existing diagnostic imaging and did not interfere with patient management or clinical decision-making.

#### 2.1.1. Inclusion Criteria

Inclusion criteria comprised pediatric esophageal radiograms acquired during routine clinical diagnostic procedures between 2021 and 2025 that were deemed diagnostically adequate by two pediatric radiology experts. Images were eligible for inclusion if they allowed expert assessment of esophageal anatomy and luminal patency, regardless of whether the esophagus was fully or partially visualized within a single radiographic frame and regardless of visible contrast presence.

#### 2.1.2. Exclusion Criteria

Radiograms were excluded if they exhibited severe motion artifacts, improper collimation, or technical deficiencies that precluded diagnostic interpretation of the esophagus. Images were also excluded in the presence of overlapping thoracic or mediastinal pathologies that obscured esophageal assessment, as well as postoperative studies displaying metallic implants or hardware that could confound interpretation.

Radiographs obtained outside the predefined study period or lacking informed consent for scientific use were also excluded.

#### 2.1.3. Image Parameters

All radiograms were obtained using standard hospital equipment under routine clinical conditions. The images were fully anonymized prior to analysis, with all personal identifiers and metadata removed in accordance with institutional data protection guidelines. No preprocessing, filtering, or contrast adjustments were applied to preserve the authenticity of diagnostic data. The X-ray images were stored in JPEG format with a native resolution ranging between approximately 2600 × 1300 px, depending on the acquisition device, while maintaining full diagnostic clarity.

### 2.2. Image Selection and Expert Review

The selection of esophageal radiograms was performed independently by two pediatric radiology experts, who reviewed all available images to identify those meeting the predefined diagnostic and technical quality standards. Only radiographs deemed clinically reliable and of sufficient image quality by both experts were included in the final dataset. The assessment process ensured that all images used in the study accurately represented the clinical categories under investigation and were free from artifacts or limitations that could hinder diagnostic interpretation.

### 2.3. Methodology: BiomedCLIP

#### 2.3.1. Model Selection and Inference Setup

In this study, we used BiomedCLIP, a pretrained vision–language foundation model derived from the CLIP (Contrastive Language–Image Pretraining) architecture, strictly in a zero-shot inference setting. Importantly, no fine-tuning, additional training, or weight adaptation was performed on the model using the pediatric esophageal radiographs analyzed in this study.

The model was obtained directly from the official public repository (BiomedCLIP-PubMedBERT_256-vit_base_patch16_224) and evaluated ‘as-is’, relying solely on its pretrained multimodal embedding space to perform text–image similarity matching. Classification decisions were generated by comparing image embeddings with predefined textual prompts representing each clinical category.

#### 2.3.2. Prompting Strategy and Methodological Constraint

For each classification task, we used a single fixed pair of prompts (one affirmative and one negated statement) and did not perform prompt ensembling, prompt search, or learned prompt tuning. Because CLIP-based vision–language models are known to be sensitive to prompt wording, this design choice constitutes a methodological limitation and may have influenced performance, particularly under conditions of class imbalance or overlapping visual semantics across tasks

This experimental design was intentionally chosen to assess the out-of-the-box generalization capability of a biomedical foundation model when applied to a rare pediatric imaging task, without any domain-specific supervision or optimization.

#### 2.3.3. Background Information on the Original BiomedCLIP Training

BiomedCLIP was originally trained by its developers on the PMC-15M dataset using a CLIP ViT-B/16 architecture combined with PubMedBERT as the text encoder. Details regarding the original training configuration, including optimizer settings, batch size, number of epochs, and hardware infrastructure, are provided in the original model publication and are not part of the experimental pipeline used in this study.

#### 2.3.4. Hardware and Software Configuration

All computational tasks were executed on a system equipped with two NVIDIA L40S GPUs, supported by 16 virtual CPUs and 124 GB RAM (96 GB VRAM), running on the RunPod platform (1181 Nixon Dr. #1158 Moorestown, NJ 08057). The software environment used a Docker image (pytorch:2.1.0-py3.10-cuda11.8.0-devel-ubuntu22.04), which includes PyTorch 2.1.0, Python 3.10, CUDA 11.8, and Ubuntu 22.04, together with the required NVIDIA CUDA libraries (including cuDNN) for training and evaluation.

#### 2.3.5. Model Architecture

BiomedCLIP is based on the Vision Transformer (ViT-B/16) architecture for the visual encoder, which splits input images into 16 × 16 patches. This architecture was selected for its capacity to capture long-range dependencies in medical images. Each patch is treated as a token and processed through a self-attention mechanism. The model processes images at a resolution of 224 × 224 pixels, although trials with higher resolution (384 × 384) indicated potential performance gains at the cost of increased training time.

ViT-B/16 contains approximately 86 million trainable parameters. To meet the demands of biomedical text processing, the default text encoder was extended from 77 to 256 tokens, enabling the handling of longer, more detailed descriptions.

#### 2.3.6. Data Processing and Evaluation

Evaluation was based on anonymized pediatric esophageal X-ray images to iteratively assess model performance across the defined classification categories. All analyses were performed using frozen pretrained weights of BiomedCLIP, without any model parameter updates. Text prompts were constructed to represent each class:

- Category 1: Esophagus Visibility
  ○ ‘This is an image of the esophagus.’
  ○ ‘This is not an image of the esophagus.’

This prompt pair queried the model about general esophageal presence or visibility within the image and did not explicitly specify complete or continuous visualization of the entire esophageal lumen. In contrast, the reference-standard annotations applied a strict clinical definition of full esophageal visibility, as described in the Study Design section.

- Category 2: Contrast Agent Presence
  ○ ‘This is an image with visible contrast agent.’
  ○ ‘This is an image without visible contrast agent.’

- Category 3: Esophageal Stricture
  ○ ‘This is an image showing esophageal stricture.’
  ○ ‘This is an image without esophageal stricture.’

### 2.4. Statistical Analysis

The objective of this analysis is to assess the performance of the BiomedClip AI (further—AI) in answering clinical yes-or-no questions based on X-ray image recognition, specifically in determining the occurrence or non-occurrence of a clinical event (full esophageal visibility, use of contrast, presence of esophageal stricture).

Additionally, the analysis aims to evaluate the reliability of the AI’s probability estimates in predicting the presence or absence of the event.

Descriptive statistics were employed to evaluate the central tendency (mean and median), variability (standard deviation), position (interquartile range), and range of the predicted probabilities for both options: occurrence and non-occurrence of the event.

To evaluate the performance of the AI, its predicted probabilities are converted into binary classifications using a threshold, such as 0.5. The values greater than or equal to this threshold, the prediction is classified as an ‘occurrence of the event’ (1); otherwise, it is classified as a ‘non-occurrence of the event’ (0).

The AI’s binary predictions are compared against the observed values (ground truth) by estimating a comprehensive set of 27 classification metrics. Metrics included measures of overall accuracy, error rate, and classification balance, as well as precision, recall and specificity to evaluate the model’s predictive performance. The balanced accuracy was calculated to account for class imbalances, and the F1-score was used to combine precision and recall into a single metric. Additional metrics, such as the geometric mean and Adjusted Geometric Mean were employed to further assess the model’s performance in the context of imbalanced data.

Calibration and agreement between predictions and observations were analyzed using Cohen’s Kappa, the Matthews correlation coefficient, and the Bias Metric Index. Metrics specific to diagnostic performance included the positive predictive value (precision), negative predictive value, false discovery rate, false omission rate, false positive rate, and false negative rate. The positive likelihood ratio, negative likelihood ratio, and diagnostic odds ratio were calculated to evaluate the diagnostic utility of the model.

The area under the receiver operating characteristic curve was used to assess the model’s ability to discriminate between cases with and without contrast. The prevalence of positive cases and the threshold prevalence were also measured to understand the dataset’s class distribution and its influence on model performance. The critical success index and the Fowlkes-Mallows Index were included to evaluate the model’s ability to balance true positive and false positive predictions. Finally, difference in predictive values was calculated as a measure of calibration bias, and the p4 metric was used as an additional evaluation of predictive performance.

The calibration curve is utilized to determine how well the predicted probabilities correspond to observed frequencies of occurrence, while the Brier score provides a quantitative measure of the accuracy of probabilistic predictions. The calibration curve was created by grouping the predicted probabilities into bins of equal width, ranging from 0.0 to 1.0, with each bin representing an interval of 0.1. For each bin, the observed frequency of occurrence was calculated as the mean of the actual outcomes within that bin, and the average predicted probability for the bin was also computed. These values were plotted to visually inspect the degree of calibration between predicted probabilities and observed outcomes.

To quantitatively measure the accuracy of the probabilistic predictions, the Brier score was calculated. This metric captures both the resolution and reliability of the model’s predictions, making it a robust indicator of calibration quality. The Brier score is defined as the mean squared error between the predicted probabilities and the actual outcomes and is expressed mathematically by (1):

(1) $$Brier\;Score=\;\frac{1}{N}\sum_{i=1}^{N}{\left(p_{i}\;-\;o_{i}\right)}^{2}$$

where *p_i_* represents the predicted probability for the *i*-th instance, *o_i_* represents the observed outcome (0 or 1) for the *i*-th instance, and *N* is the total number of instances.

#### 2.4.1. Characteristics of the Statistical Tool

Analyses were conducted using the R Statistical language (version 4.3.3) [31] on Windows 11 pro 64 bit (build 22631), using the packages metrica (version 2.1.0) [32], report (version 0.5.8) [33], ggplot2 (version 3.5.0) [34], and dplyr (version 1.1.4) [35].

#### 2.4.2. Characteristics of the Sample

A total of 143 pediatric esophageal X-ray images were evaluated in this study. The characteristics of the analyzed variables, annotated by two independent pediatric radiology experts serving as the reference standard, are summarized in Table 1.

The results presented in Table 1 examine three distinct scenarios. The first scenario addresses a situation in which no events occur, facilitating a baseline understanding of AI predictions in the absence of any events. The second scenario focuses on the AI predictions with the predominantly events occurrence. Finally, the third scenario considers the AI predictions when the number of events is limited.

The variable ‘full esophageal visibility’ was operationally defined in a deliberately strict manner, requiring uninterrupted visualization of the entire esophageal lumen from the upper esophageal sphincter to the gastroesophageal junction within a single radiographic frame.

This definition was applied exclusively at the reference-standard level and did not correspond directly to the textual prompt used for model inference, which queried general esophageal visibility rather than complete anatomical continuity. Under this expert-defined criterion, none of the analyzed images met the threshold for positive classification, reflecting known limitations of routine pediatric esophageal radiography rather than a data acquisition error.

## 3. Results

### 3.1. Analysis of AI Predictions for Full Esophageal Visibility on X-Ray Images

This analysis reflects a mismatch between a broad prompt formulation and a highly restrictive reference-standard definition, which resulted in a dataset with zero positive cases for full esophageal visibility.

#### 3.1.1. Descriptive Statistics and Classification Performance

The model consistently assigned low probabilities to the full-visibility class (mean 0.091, median 0.066; IQR 0.042–0.115) and high probabilities to the non-visibility class (mean 0.909, median 0.934; IQR 0.885–0.958), indicating a strong tendency to predict non-occurrence. At the 0.5 threshold, all images were classified as negative (Table 2), yielding perfect negative-class accuracy and specificity. Because no positive reference cases were present, metrics dependent on true positives—such as sensitivity, precision, and F1-score—are not informative and are therefore reported only for completeness.

#### 3.1.2. Calibration Analysis

**Figure 3:**
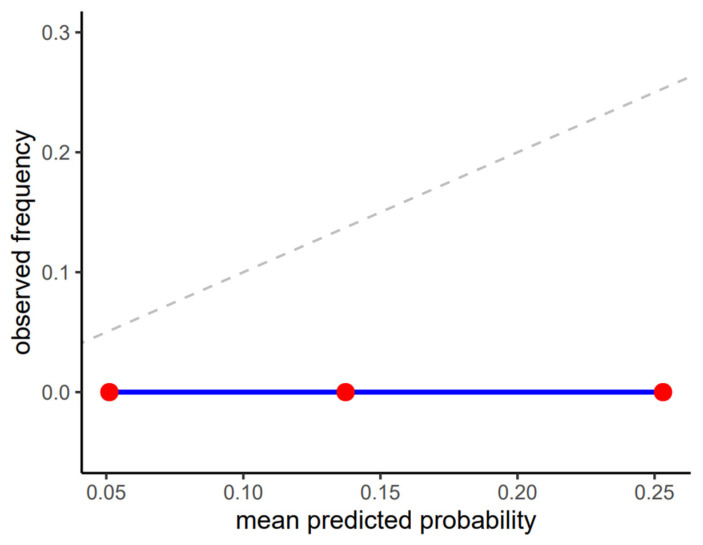
Calibration plot of predicted probabilities vs. observed frequencies of full esophageal visibility on X-ray images.

Calibration results must be interpreted cautiously in this setting. The absence of positive outcomes led to a flat calibration curve at zero across all probability bins (Figure 3) and renders the Brier score (0.013) largely prevalence-driven rather than reflective of true probabilistic reliability. Although the model assigned low to moderate probabilities to the positive class, these did not correspond to observed events in the dataset, highlighting the limited interpretability of probability estimates under extreme class imbalance.

### 3.2. Analysis of AI Predictions for Full Esophageal Visibility on X-Ray Images

#### 3.2.1. Descriptive Statistics and Overall Performance

**Figure 4:**
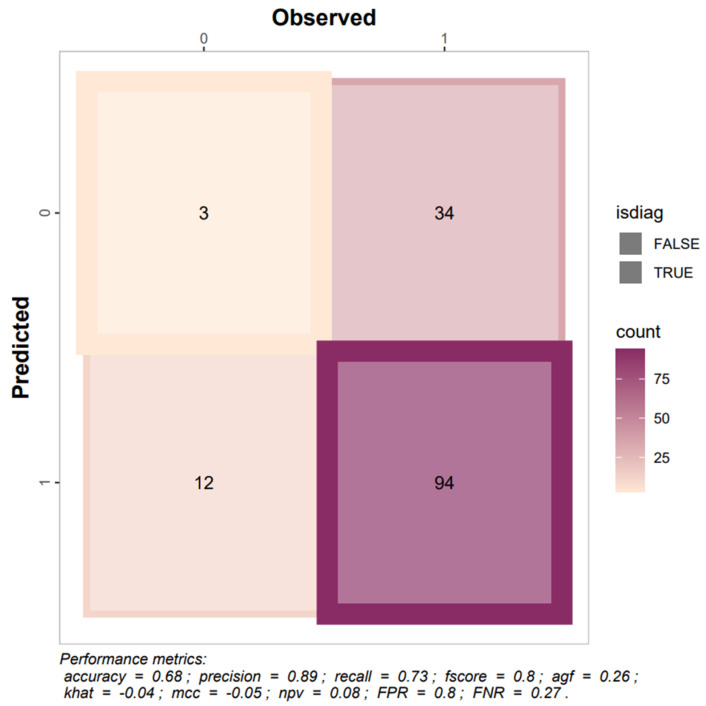
Confusion matrix depicting the AI model’s performance in predicting contrast visibility in X-ray images with reporting results of selected metrics.

For contrast detection, the model assigned moderate probabilities to the positive class (mean 0.552, median 0.556; IQR 0.494–0.635) and complementary values to the negative class (mean 0.448, median 0.444; IQR 0.365–0.505). At the 0.5 threshold, BiomedCLIP achieved an overall accuracy of 67.8% with high precision (0.887) and moderate recall/sensitivity (≈0.73–0.75), yielding an F1-score of 0.803 and good discriminative ability (AUC = 0.854; Figure 4; Table A1). These results indicate that positive predictions were usually correct and that many true contrast cases were detected.

#### 3.2.2. Limitations in Negative-Class Prediction and Calibration

**Figure 5:**
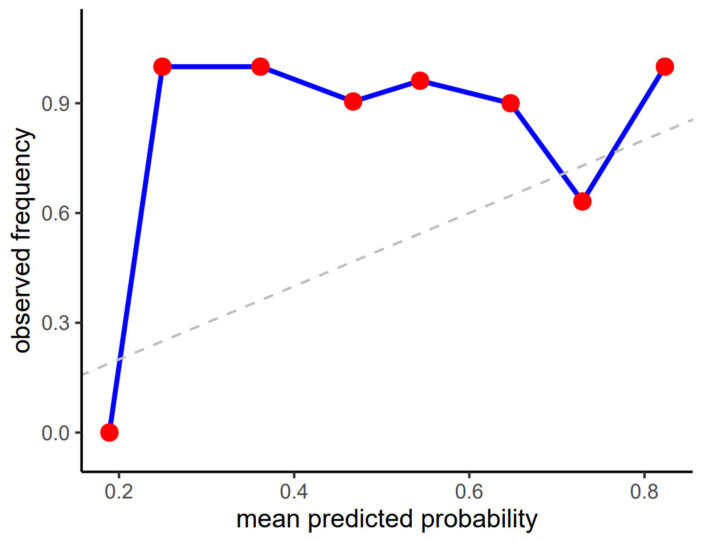
Calibration plot of predicted probabilities vs. observed frequencies of contrast visibility on X-ray images.

Despite these strengths, performance for non-contrast cases was poor. Specificity was only 0.200 with a false-positive rate of 0.800, and the negative predictive value was 0.081, indicating a strong tendency to overpredict contrast in an imbalanced dataset. Agreement metrics were correspondingly low or negative (Kappa= −0.04; MCC= −0.046), and diagnostic likelihood ratios did not support clinical usefulness. Calibration analysis revealed substantial misalignment between predicted probabilities and observed frequencies (Figure 5), particularly at the extremes of the probability range, and the Brier score of 0.242 further reflects limited reliability of probability estimates in this zero-shot setting.

Overall, BiomedCLIP demonstrated reasonable ability to identify the presence of contrast but performed poorly in recognizing its absence. The combination of low specificity, poor calibration, and class imbalance-driven bias toward positive predictions substantially limits clinical applicability and indicates a need for improved calibration strategies and task-specific adaptation.

### 3.3. Analysis of AI Predictions for Full Esophageal Stricture Visibility on X-Ray Images

#### 3.3.1. Overall Performance

**Figure 6:**
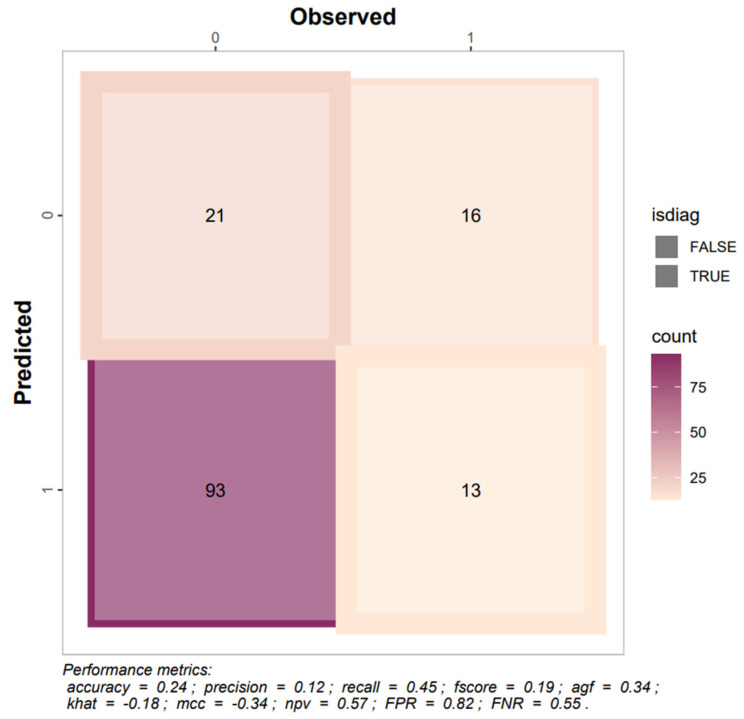
Confusion matrix depicting the AI model’s performance in predicting esophageal stricture visibility in X-ray images with reporting results of selected metrics.

BiomedCLIP performed poorly in detecting esophageal strictures and did not demonstrate clinically useful discrimination. Overall accuracy was 0.24 with low sensitivity (≈0.44) and specificity (0.18), and precision was only 0.12, indicating that most positive predictions were incorrect (Figure 6; Table A2). Agreement and correlation metrics were negative (Kappa = −0.18; MCC = −0.34), and the AUC of 0.26 indicates performance worse than random guessing.

#### 3.3.2. Calibration Analysis

**Figure 7:**
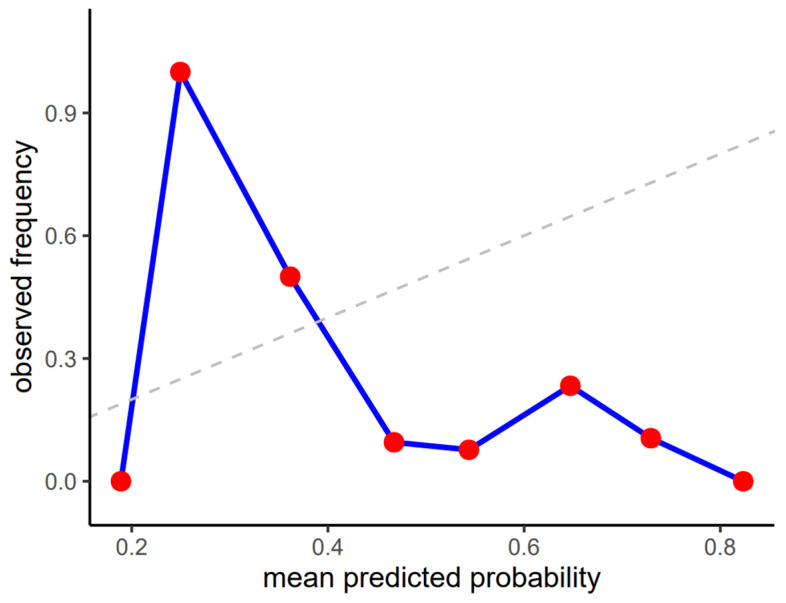
Calibration plot of predicted probabilities vs. observed frequencies of esophageal stricture visibility on X-ray images.

Calibration curves showed marked inconsistency between predicted probabilities and observed frequencies across the full probability range (Figure 7), particularly in the intermediate and high ranges, and the Brier score of 0.36 further reflects poor probabilistic reliability in this zero-shot setting.

Low sensitivity raises concerns about missed strictures, whereas very low specificity implies frequent false-positive findings that could lead to unnecessary investigations. Together with poor agreement metrics and lack of meaningful discrimination, these results indicate that the model is not suitable for clinical use in its current form.

## 4. Discussion

The aim of this study was to evaluate the ability of BiomedCLIP to classify pediatric esophageal radiographs into three clinically relevant categories: presence of contrast, full esophageal visibility, and occurrence of strictures. Based on the predefined hypotheses (H1 and H2), we assessed whether the model could reliably recognize diagnostic features essential in radiological practice.

Several of the mechanistic interpretations proposed below should be regarded as working hypotheses rather than confirmed explanations. Because this study was not designed to isolate specific causal factors underlying model behavior (e.g., feature attribution, cross-task correlations, or controlled ablation analyses), the interpretations offered here remain provisional and are intended to guide future investigations rather than establish definitive mechanisms.

The results revealed substantial differences in performance across the individual tasks, highlighting both the potential and the limitations of applying a general-purpose biomedical foundation model to rare and clinically nuanced pediatric imaging scenarios. These findings allow several important conclusions to be drawn regarding model behavior, sources of error, and directions for future research.

### Verification of Hypothesis H1: Contrast Detection Performance

Our results partially confirmed H1 (‘BiomedCLIP will accurately detect the presence of contrast’). The model achieved high precision (~88.7%) and a moderately good sensitivity (73.4–75.2%), with an AUC of 85.4%, indicating decent discriminative ability for distinguishing contrast-enhanced images from non-contrast images. For context, dedicated CNN models have achieved near-perfect performance (AUC ~0.98–1.0) on detecting intravenous contrast in CT scans, so while BiomedCLIP’s AUC is lower, it still suggests the model learned relevant features [36]. However, the critical issue was extremely low specificity (~20%) and a very high false positive rate (FPR ~80%), meaning the model frequently predicted contrast when none was present. This behavior can be largely attributed to the severe class imbalance in our data (≈89.5% of images had contrast). The model was biased toward the dominant class, over-predicting ‘contrast present’ for most inputs. Such effects of imbalance have been noted in the literature—for example, Zhang et al. observed that a model trained on skewed data ended up classifying all test images as positive, essentially learning spurious dataset-specific cues instead of the true signal [37]. In our case, the high prevalence of contrast-enhanced images may have encouraged BiomedCLIP to rely on nonspecific brightness cues when predicting the presence of contrast, potentially contributing to the large number of false-positive classifications.

Another factor is the model’s poor probabilistic calibration. We found that BiomedCLIP’s confidence scores were not well-aligned with reality: it underestimates probabilities for truly non-contrast cases and grossly overestimates in many supposed ‘contrast’ cases. This lack of calibration indicates the model’s predicted probabilities (confidence) did not reflect the actual likelihood of contrast being present. Such miscalibration is a known issue in deep learning models [38]. Proper calibration—for instance, via Platt scaling or isotonic regression—can reduce the mismatch between the model’s confidence and real-world frequencies [38]. The fact that BiomedCLIP was not fine-tuned on a well-balanced, task-specific dataset (e.g., esophagus or GI studies with and without contrast) likely exacerbated this issue.

Furthermore, BiomedCLIP’s broad pretraining may have led to an overly general interpretation of radiographic features. In this scenario, the model may misinterpret normal high-density structures (such as air–fluid interfaces or dense anatomy) as contrast material. Foundation models like CLIP are known to sometimes learn false correlations, which hurts performance when the data distribution changes [9]. Here, features common in contrast-enhanced scans (e.g., bright blood vessels) might also appear in non-contrast scans due to other factors, confusing the model. Indeed, prior work has noted that BiomedCLIP can produce false-positive findings on normal images in zero-shot settingsarxiv.org. Park et al. reported that without specialized prompting or adaptation, BiomedCLIP’s attention maps highlighted many regions in a normal image as if they were abnormalities [39]. This suggests that our model, lacking fine-tuning for the specific context, was prone to interpreting any intense signal as contrast, even when it was just normal anatomy or artifact.

In summary, BiomedCLIP did well in flagging actual contrast usage (high precision and decent sensitivity), but it struggled to recognize true negatives. The overwhelming false positive rate (80%) meant that ‘no contrast’ predictions were almost always wrong (NPV only ~8%). In practical terms, such a tool would inundate clinicians with false alarms. Low specificity greatly limits clinical utility—an AI model that cries wolf too often can erode user trust and burden healthcare providers. Excessive false alerts increase workload and can lead to alarm fatigue [40]. In fact, prior studies note that AI systems with very low specificity are essentially untenable in clinical practice [41]. Thus, despite BiomedCLIP’s promise in detecting contrast when present, its inability to reliably identify absence of contrast severely undermines its clinical usefulness.

The BiomedCLIP model’s failure to detect esophageal strictures is evident from its poor metrics (accuracy ~24%, sensitivity ~44%, specificity ~18%, precision ~12%), with an AUC of only 0.26 and negative MCC/Kappa. These values indicate that the model predicts strictures worse than random guessing, implying a systematic misclassification rather than isolated errors. Literature on foundation vision–language models suggests a potential explanation: the model may not have developed a robust representation of the visual concept of ‘esophageal stricture’. General-purpose or broadly trained models often rely on salient cues present in training data and may miss subtle, domain-specific features [42]. In our case, BiomedCLIP may have been disproportionately influenced by the conspicuous presence of contrast medium in esophagrams—a feature common to both stricture and non-stricture cases—rather than by the more subtle morphological signs of luminal narrowing. This aligns with reports that BiomedCLIP and similar biomedical CLIP models, even though trained on millions of image–text pairs, show only moderate success on specialized radiographic tasks and can be outperformed by models trained on task-focused data [3]. In other words, without explicit examples of strictures during training, the model defaulted to an ‘abnormal X-ray’ heuristic (e.g., seeing contrast as an abnormality), rather than truly recognizing the lesion.

Moreover, esophageal strictures present very subtle radiographic changes—slight narrowing of the barium column, irregular edges, or mild proximal dilatation—which are challenging even for experts and require careful analysis of edges and contours. Vision transformers like those in CLIP were not specifically trained to detect such fine-grained structural details. Prior studies note that deep models struggle with blurred or indistinct boundaries that radiologists discern using anatomical knowledge [43]. The small number of positive stricture examples (*N* = 29) further exacerbates this issue. Foundation models excel when given large and diverse training data, but in a zero-shot setting with few or no examples of a rare pathology, the model cannot derive a novel diagnostic concept without exposure to representative examples [43]. Indeed, research on anomaly detection using CLIP indicates that a vanilla pre-trained model has limited zero-shot ability to identify novel anomalies without task-specific adaptation, highlighting the necessity of fine-tuning or prompt optimization [44]. In our study, BiomedCLIP had neither abundant stricture samples nor a fine-tuning step, so it simply could not learn the subtle pattern of a stricture.

Another possible contributing factor is feature interference between categories. The presence of contrast medium—a visually salient radiographic signal—may have dominated the model’s predictions at the expense of subtler pathology-related cues. In other words, the model confuses ‘contrast present’ with ‘stricture present’, since both appear as high-intensity regions on X-ray. Without any adversarial training or explicit negative examples to disentangle these factors, such spurious correlations can mislead the modelarxiv.org [45]. Recent work on medical CLIP models has shown that, if two conditions share visual similarities, the model will often merge them unless explicitly taught otherwise. The lack of counter-examples (e.g., images with contrast but no stricture) in training means the model never learned to differentiate those scenarios. As a result, it treats any vivid contrast outline as pathology, yielding many false positives and negatives. This phenomenon is consistent with the observation that fine-grained labels in medical imaging can confuse contrastive models—false negative pairings during training (e.g., treating a stricture image vs. a contrast-only image as unrelated) can misdirect the learned representations [46]. In summary, BiomedCLIP may not have learned to reliably distinguish between a normal contrast esophagram and one with a subtle stricture and may instead have relied on the more salient feature of contrast presence, which alone is not diagnostic of stricture.

It is instructive to compare these zero-shot results with dedicated, supervised approaches from the literature. When ample training data and task-specific modeling are applied, performance improves dramatically. For example, Togo et al. developed a deep CNN for detecting gastritis on barium radiographs and achieved ~96% sensitivity and 98% specificity [47]. Similarly, a recent two-stage AI system for esophageal cancer on barium swallow images showed excellent accuracy by first locating abnormal regions and then classifying them (in essence, learning the exact features of the lesion. These successes underscore that recognizing esophageal lesions is feasible for AI, but requires training on the specific visual signatures of the pathology. In contrast, our BiomedCLIP model was a general biomedical image-language model applied in a zero-shot manner, without ever having seen a labeled esophageal stricture example. The literature consistently emphasizes that such foundation models must be adapted or fine-tuned for fine-grained medical tasks to avoid misidentifying correlated features as disease [3,42,45]. In light of our results and prior studies, the conclusion is that Hypothesis H2 is not supported because BiomedCLIP lacked the specialized learning needed to distinguish a stricture from look-alike scenarios, leading it to perform worse than random guessing in stricture detection.

Given the extremely poor performance metrics observed for esophageal stricture detection, additional control considerations were evaluated to exclude trivial implementation errors. The consistency of label assignment and prompt polarity was verified, and no evidence of label inversion or prompt asymmetry effects was identified. The observed performance, therefore, appears to reflect genuine model behavior rather than an obvious technical artifact.

While the hypothesis that semantic overlap between contrast presence and stricture appearance may contribute to model confusion is plausible, it should be interpreted as a working hypothesis rather than a confirmed mechanism. The present study was not designed to perform dedicated cross-task correlation or subgroup analyses, and such investigations represent an important direction for future work.

Qualitative inspection of model outputs suggested that predicted probabilities for esophageal stricture frequently mirrored those obtained for contrast presence, even in cases without expert-confirmed strictures. This observation is consistent with the possibility that the model relies on salient contrast-related intensity cues rather than subtle morphological features of luminal narrowing. However, this interpretation remains descriptive and requires targeted quantitative validation in future studies.

This study has several limitations that should be acknowledged. First, the dataset exhibited significant class imbalance, particularly with regard to esophageal strictures, which were present in only 29 of 143 cases. This low prevalence likely hindered the model’s ability to learn or infer meaningful representations for this critical class. Second, BiomedCLIP was applied in a zero-shot setting without any task-specific fine-tuning. While this reflects a common use case for foundation models, it limits their ability to capture nuanced domain-specific features necessary for clinical accuracy. Third, the model was not pre-trained on pediatric or esophageal imaging data, which may have restricted its capacity to recognize anatomical patterns specific to the population and pathology under investigation. Additionally, the observed semantic overlap between contrast-enhanced images and stricture cases suggests the model may rely more on visual saliency (e.g., brightness from contrast) than on true structural abnormalities, leading to category interference. Fourth, the evaluation was based on a relatively small, single-center dataset, which limits generalizability. Finally, no external validation was performed on an independent cohort, preventing robust assessment of the model’s performance across diverse populations or imaging protocols. These limitations underscore the need for larger, balanced, and clinically annotated datasets as well as fine-tuning strategies tailored to specific diagnostic tasks in future research.

A key methodological limitation of this study is the simplicity of the text prompt design used for zero-shot inference. For each classification task, we applied only a single pair of fixed prompts per class (e.g., ‘This is an image with visible contrast agent’ vs. ‘This is an image without visible contrast agent’) without evaluating alternative wordings, prompt templates, or prompt ensembling. Because CLIP-based vision–language models are known to be sensitive to prompt phrasing, the use of only one prompt formulation may have constrained performance and contributed to unstable behavior, particularly under class imbalance and in the presence of overlapping visual semantics (e.g., contrast-related brightness cues). Future work should systematically test multiple prompt variants, incorporate clinically grounded phrasing (e.g., ‘contrast-filled esophageal lumen’, ‘anastomotic narrowing with proximal dilatation’), and evaluate prompt ensembling or learned prompt tuning to reduce wording dependence and improve robustness.

The discrepancy between the generic prompt used for esophageal visibility (‘This is an image of the esophagus’) and the strict reference-standard definition of full esophageal visualization highlights an important limitation of zero-shot vision–language inference. While the model was queried about general esophageal presence, expert annotations required complete anatomical continuity, a condition not achievable in routine pediatric radiography. This mismatch likely contributed to non-zero probability outputs despite the absence of positive ground-truth cases and illustrates the sensitivity of zero-shot models to semantic granularity in prompt design.

Because the reported probabilities were derived from normalized similarity scores rather than calibrated posterior estimates, calibration metrics should be interpreted as reflecting relative model confidence rather than true clinical probability.

## 5. Conclusions

This study evaluated the zero-shot diagnostic performance of BiomedCLIP in classifying pediatric esophageal radiographs into three clinically relevant categories: presence of contrast agent, full esophageal visibility, and esophageal stricture. BiomedCLIP demonstrated moderate discriminatory ability for contrast detection (AUC 0.854) and high precision; however, its very low specificity and high false-positive rate substantially limit reliability. Importantly, even if improved, automated identification of contrast use has limited direct clinical utility because it is typically straightforward for clinicians and does not itself constitute a diagnostic endpoint. The model was unable to detect esophageal strictures with acceptable performance (AUC 0.26, low sensitivity and specificity), indicating that, in its current zero-shot form, BiomedCLIP is not suitable for supporting clinical decisions in postoperative esophageal atresia follow-up. Overall, our findings suggest that foundation vision–language models may recognize high-salience imaging cues (e.g., contrast-related intensity patterns), but they fail to capture subtle morphologic abnormalities such as strictures without domain-specific adaptation. Future work should prioritize task-specific fine-tuning on balanced datasets, improved prompt strategies (including prompt ensembles), and methods that explicitly disentangle contrast-related features from pathology-related cues to increase interpretability and clinical relevance.

## Figures and Tables

**Table 1:** The distribution of the observed events.

Study Trait	N	Events ^a^
Full esophageal visibility	143	0 (0%)
Presence of contrast in the esophagus	143	128 (89.5%)
Esophageal stricture	143	29 (20.3%)
^a^ *n* (%)		

Note: N—sample size; n—group size.

**Table 2:** Confusion matrix for AI model predictions on non-occurrence of full esophageal visibility.

AI model Predictions	Observed Data	
	Non-Visible	Visible
Non-visible	143 (100%)	0
Visible	0	0

## Data Availability

The data are contained within the article.

## Abbreviations

The following abbreviations are used in this manuscript:

ZSL	Zero-shot Learning
OSAIM	Open-source Artificial Intelligence Models
CLIP	Contrastive Language–Image Pretraining Model

## Appendix A. Classification Performance Metrics Results

**Table A1:** Comprehensive performance metrics for the AI model in detecting contrast visibility on X-ray images.

Metric	Value	95% CI
Accuracy	0.678	[0.601, 0.748]
Error rate	0.322	[0.252, 0.399]
Precision	0.887	[0.861, 0.916]
Recall (sensitivity)	0.734	[0.656, 0.812]
Specificity	0.200	[0.000, 0.400]
Balanced accuracy	0.467	[0.367, 0.581]
F1-score (fscore)	0.803	[0.748, 0.852]
Adjusted geometric mean (agf)	0.264	-
Geometric mean	0.383	[0.000, 0.556]
Cohen’s Kappa (khat)	−0.04	[−0.169, 0.096]
Matthews’s correlation coefficient (mcc)	−0.05	[−0.188, 0.110]
Fowlkes-Mallow’s index	0.807	[0.755, 0.853]
Bayes metric index	−0.066	[−0.266, 0.162]
Critical success index	0.862	[0.597, 0.743]
Difference in predictive values	−0.032	[−0.136, 0.077]
Positive likelihood ratio	0.917	[0.727, 1.276]
Negative likelihood ratio	1.328	[0.605, 1.990]
Diagnostic odds ratio	0.691	[0.007, 2.073]
Negative predictive value (npv)	0.081	[0.000, 0.162]
False positive rate (FPN)	0.800	[0.600, 1.000]
False negative rate (FNR)	0.266	[0.188, 0.344]
False discovery rate	0.113	[0.084, 0.139]
False omission rate	0.919	[0.838, 1.000]
Prevalence	0.895	[0.834, 0.935]
Prevalence threshold	0.343	[0.242, 0.473]
The area under the receiver operating characteristic curve	0.854	[0.179, 0.521]
Additional evaluation of predictive performance (P4)	0.202	[0.066, 0.412]

**Table A2:** Comprehensive performance metrics for the AI model in detecting esophageal stricture visibility on X-ray images.

Metric	Value	95% CI
Accuracy	0.24	[0.17, 0.31]
Error rate	0.76	[0.69, 0.83]
Precision	0.12	[0.06, 0.19]
Recall (sensitivity)	0.45	[0.27, 0.63]
Specificity	0.18	[0.11, 0.26]
Balanced accuracy	0.32	[0.22, 0.42]
F1-score (fscore)	0.19	[0.11, 0.29]
Adjusted geometric mean (agf)	0.34	-
Geometric mean	0.29	[0.18, 0.39]
Cohen’s Kappa (khat)	−0.18	[−0.29, −0.07]
Matthews’s correlation coefficient (mcc)	−0.34	[−0.47, −0.21]
Fowlkes-Mallow’s index	0.23	[0.14, 0.33]
Bayes metric index	−0.37	[−0.56, −0.18]
Critical success index	0.10	[0.05, 0.17]
Difference in predictive values	−0.31	[−0.46, −0.16]
Positive likelihood ratio	0.55	[0.36, 0.83]
Negative likelihood ratio	3.00	[1.91, 4.71]
Diagnostic odds ratio	0.18	[0.09, 0.37]
Negative predictive value (npv)	0.57	[0.38, 0.74]
False positive rate (FPN)	0.82	[0.74, 0.89]
False negative rate (FNR)	0.55	[0.37, 0.73]
False discovery rate	0.88	[0.81, 0.94]
False omission rate	0.43	[0.26, 0.62]
Prevalence	0.20	[0.14, 0.27]
Prevalence threshold	0.39	[0.22, 0.49]
The area under the receiver operating characteristic curve	0.26	[0.17, 0.35]
Additional evaluation of predictive performance (P4)	0.23	[0.16, 0.29]
